# Do Adverse Intrauterine Environments Affect Body Mass Index in Infants and Preschool Children?

**DOI:** 10.1002/ajhb.70359

**Published:** 2026-10-30

**Authors:** Gabriela Koglin, Clécio Homrich da Silva, Denise Ruschel Bandeira, Nadia Cristina Valentini, Marcelo Zubaran Goldani, Juliana Rombaldi Bernardi

**Affiliations:** ^1^ Graduate Program in Child and Adolescent Health Universidade Federal do Rio Grande do Sul Porto Alegre Brazil; ^2^ Hospital de Clínicas de Porto Alegre Porto Alegre Brazil; ^3^ Graduate Program in Psychology Universidade Federal do Rio Grande do Sul Porto Alegre Brazil; ^4^ Graduate Program in Human Movement Science Universidade Federal do Rio Grande do Sul Porto Alegre Brazil; ^5^ Graduate Program in Food, Nutrition and Health Universidade Federal do Rio Grande do Sul Porto Alegre Brazil

**Keywords:** body mass index, developmental origins of health and disease, high‐risk pregnancy, longitudinal study, prenatal exposure delayed effects

## Abstract

**Introduction:**

Exposure to adverse intrauterine environments resulting from different maternal clinical conditions may alter childhood growth patterns. This study investigated the influence of such environments on body mass index‐for‐age (BMI‐for‐age) trajectories from birth to preschool age.

**Methods:**

A total of 342 mother–child dyads recruited from three hospitals in southern Brazil were allocated into five groups: diabetes mellitus (DM), systemic arterial hypertension (SAH), maternal smoking, small for gestational age (SGA, used here as a proxy for impaired fetal growth), and a control group. Children were assessed at seven time points from birth to preschool age using anthropometric measurements to calculate BMI‐for‐age z‐scores.

**Results:**

Generalized estimating equations (GEE) identified a significant interaction between group and time (*p* < 0.001). Among girls, at birth, the DM group presented higher BMI‐for‐age z‐scores than the smoking group and reached elevated BMI‐for‐age z‐scores at preschool age, differing from the control group. At 6 months of age, girls in the SGA group showed a marked increase in BMI‐for‐age. Among boys, the SGA group reached higher BMI‐for‐age values at preschool age than the DM and control groups. Differences were observed across time, group, and sex in the SGA and control groups (*p* < 0.001).

**Conclusion:**

These findings suggest that adverse intrauterine environments may influence BMI‐for‐age trajectories during early life, with greater variability and evidence of catch‐up growth among children exposed to maternal DM and SGA. Results should be interpreted cautiously due to unequal subgroup sizes and substantial loss to follow‐up at preschool age.

## 1 Introduction

There is evidence that a child's growth pattern during the first years of life is strongly influenced by fetal growth, which may increase the likelihood of adverse metabolic outcomes when the fetus is exposed to an adverse intrauterine environment (Casirati et al. 2022; Silveira et al. 2007). Multifactorial diseases during pregnancy, such as diabetes mellitus (DM) and systemic arterial hypertension (SAH), result from complex interactions between genetic and environmental factors that act from the fetal period and may determine the adult phenotype (Casirati et al. 2022). In addition, other common gestational clinical conditions with known repercussions for the fetus include maternal smoking and intrauterine growth restriction (IUGR).

Hyperglycemia caused by DM during pregnancy has been associated with poorer outcomes in the offspring (Bianco and Josefson 2019; Burlina et al. 2019; Li et al. 2025) and with childhood adiposity (Rezende 2014). Children born to mothers with DM present increased rates of cardiovascular disease (CVD) from childhood through early adulthood (< 40 years) (Yu et al. 2019). Chronic hypertension is associated with fetal morbidity in the form of IUGR and with maternal morbidity, manifested as increased blood pressure (Roberts et al. 2013). The intrauterine environment resulting from hypertensive disorders may establish a specific fetal context, leading to programming of organ function and metabolism, with an important impact on child health (Pinheiro et al. 2016).

It is well established that newborns of mothers who smoke are, on average, smaller than those born to non‐smokers (Einarson and Riordan 2009; Rogers 2019), with an increased risk of overweight or obesity during adolescence (Perkins et al. 2023) and an increased risk of insulin resistance, Type 2 DM, and SAH (Behl et al. 2013; Rogers 2019). This may be explained by fetal hypoxia resulting from carbon monoxide exposure, nicotine‐induced reduction in placental blood flow, as well as placental toxicity or growth‐related effects caused by the thousands of chemical compounds present in cigarette smoke (Rogers 2019; Talhout et al. 2011). Similarly, newborns and infants born small for gestational age (SGA), many of whom may have experienced impaired fetal growth, tend to receive clinical attention aimed at promoting weight gain; however, rapid postnatal weight gain has been associated with an increased incidence of cardiovascular diseases and obesity in adulthood (Barker 1995; Calcaterra et al. 2023; Silveira et al. 2007).

Considering the effects of adverse intrauterine environments resulting from different maternal clinical conditions on metabolic programming and the risk of developing chronic diseases, this study aimed to investigate their influence on child growth, assessed by BMI‐for‐age, in boys and girls from birth to preschool age.

## 2 Methods

This cohort is part of the IVAPSA project, whose Phase I (2011–2016) was entitled “Impact of Variations in the Perinatal Environment on Newborn Health in the First Six Months of Life” (Bernardi et al. 2012), and whose Phase II (2017–2019) was entitled “Impact of Variations in the Perinatal Environment on Child Health in the First Five Years of Life.”

In the first phase of the project, a convenience sample of postpartum women with recent delivery (24–48 h before the interview) and their live newborns was recruited. Mothers who had delivered infants between 37 and 42 weeks of gestation were invited to participate. Mother–child dyads were allocated into five groups defined based on maternal clinical conditions occurring during pregnancy, and their different adverse intrauterine environments, or even pre‐existing before gestation:

1. DM: postpartum women diagnosed with Type 1, Type 2, or gestational DM and their offspring;
2. SAH: postpartum women diagnosed with hypertensive disorders, including pre‐eclampsia, eclampsia, pre‐eclampsia superimposed on chronic hypertension, chronic SAH, or gestational hypertension, and their offspring;
3. Maternal smoking (smokers): postpartum women who answered affirmatively to smoking during pregnancy at the first interview and their offspring. Detailed information on smoking intensity, trimester‐specific exposure, smoking cessation during pregnancy, and cumulative tobacco exposure was not consistently available in the cohort database; therefore, smoking was analyzed as a binary exposure variable (yes/no).
4. SGA defined impaired fetal growth: term newborns classified as SGA, defined as birth weight below the 5th percentile according to the Alexander growth curve parameters (Alexander et al. 1996); because serial prenatal growth assessments and fetal Doppler data were not available, pathological fetal growth restriction could not be formally diagnosed. Therefore, this group should be interpreted as an SGA‐defined proxy for impaired fetal growth rather than as a definitive diagnosis of IUGR;
5. Control: postpartum women who did not present any of the above conditions and their offspring.

The inclusion criteria for IVAPSA Phase I were postpartum women who delivered at one of the three hospitals previously described and who resided in the municipality of Porto Alegre, Rio Grande do Sul, Brazil. Exclusion criteria were HIV‐positive mothers, preterm newborns, twins, or those with congenital malformations or early hospitalization, as these conditions could potentially influence child growth and development during the first 6 months of life and their future health and disease trajectories. For IVAPSA Phase II, all mother–child dyads that had participated in the interview conducted at the child's sixth month of life were eligible for inclusion.

Participants were recruited from the rooming‐in units of three public hospitals in Porto Alegre, the capital of Rio Grande do Sul, in southern Brazil. Mothers and newborns presenting more than one clinical condition were excluded from the analysis. More detailed information regarding the interviews, instruments, and assessments used in the IVAPSA project (Phases I and II) can be found in previous publications (Bernardi et al. 2012; Werlang et al. 2019).

Seven assessments were conducted: within the first 48 h after birth, at 7 and 15 days, at 1, 3, and 6 months postpartum, and at preschool follow‐up. For the preschool assessment, chronological age was calculated in completed months from the date of birth to the date of assessment and analyzed as a continuous variable in months. In this study, the term “preschool age” refers to children assessed during Phase II, before school entry and between 3 and 5 years of age, with reference to Level 0 (Early Childhood Education) of UNESCO's International Standard Classification of Education (OECD, Eurostat, & UNESCO Institute for Statistics 2015). Assessments conducted at the 1st and 6th months and at preschool age took place at the Clinical Research Center (CRC) of HCPA, whereas the remaining assessments were carried out at the participants' homes, except the first assessment, which was conducted in the rooming‐in unit at the hospitals.

In Phase I of the IVAPSA project, during the first postpartum interview and after signing the Written Informed Consent Form (ICF), a questionnaire was administered to collect prenatal information (including pre‐pregnancy weight recorded in the prenatal care card) and perinatal information (newborn sex, Apgar score, mode of delivery, and birth weight, obtained from hospital records), as well as socioeconomic, demographic, and biological data (maternal age and skin color/race, marital status, educational level, family income, and parity). Each mother–child dyad was assigned a unique identification number corresponding to the questionnaires and assessments performed, thereby ensuring participant anonymity. In Phase II of the IVAPSA project, parents or legal guardians signed a new ICF, and information on the total duration of human breastfeeding was assessed.

Losses to follow‐up were defined as missed data collection at any study time point or the absence of data required for the research, such as incomplete self‐administered questionnaires. However, loss to follow‐up did not imply definitive exclusion of the mother–child dyad from the study, as active attempts were made to interview at subsequent visits. Refusals were defined as mothers who, after personal or telephone contact, explicitly stated that they were no longer interested in participating in the research. Following this explicit refusal, the dyad was excluded from further interviews.

Sample size calculation was originally performed using WinPepi software (version 11.44), estimating 72 mother–child pairs per exposure group and twice this number for the control group (Bernardi et al. 2012). However, in the present study, the number of participants in each exposure group was determined by the natural occurrence of maternal conditions within the cohort, rather than by the original sample size assumptions. As a result, the findings should be interpreted with caution and considered exploratory, particularly for the SAH and SGA‐defined impaired fetal growth groups, which had smaller sample sizes and consequently reduced statistical power to detect small‐to‐moderate differences.

During follow‐up interviews, children's weight and length were measured in duplicate according to a standardized protocol (Lohman et al. 1988). The Anthro (version 3.2.2) and AnthroPlus (version 1.0.4) software programs were used to calculate and standardize the BMI‐for‐age indicator as z‐scores. Maternal pre‐pregnancy BMI was calculated according to World Health Organization recommendations (Institute of Medicine 2009).

Quantitative variables were described as means and standard deviations for continuous parametric variables, and as medians and interquartile ranges for non‐parametric variables. Categorical variables were described as absolute and relative frequencies. The Shapiro–Wilk test was used to assess normality.

For comparisons among the study groups (DM, SAH, smokers, SGA, and control), one‐way analysis of variance (ANOVA) was used for normally distributed data, followed by post hoc testing with Bonferroni correction. When the assumptions of normality were not met, the non‐parametric Kruskal‐Wallis test was applied, with multiple comparisons performed using Dunn's test with Bonferroni adjustment. Categorical variables were analyzed using Pearson's *χ* ^2^ test, together with the analysis of adjusted residuals.

Due to the availability of children's anthropometric data (weight, length/height, and BMI) at seven time points (birth; 7, 15, and 30 days; 3 and 6 months; and preschool age), it was possible to evaluate their growth trajectories. For the longitudinal analysis of BMI‐for‐age z‐scores across follow‐up time points, we used a model estimated by generalized estimating equations (GEE), assuming a normal/Gaussian distribution for the response variable (BMI‐for‐age z‐score), complemented by the Least Significant Difference (LSD) test. The model was adjusted for age, education, parity, and maternal pre‐pregnancy BMI; marital status; family income; mode of delivery; birth weight, and child sex. These covariates were selected because they differed significantly between groups and are described in the literature as potential confounding factors. Given the unequal group sizes and the limited number of participants in some exposure categories, especially at preschool follow‐up, the GEE approach was chosen because it allows the use of repeated measurements available for each child without requiring complete data at all time points. Nevertheless, precision is lower in the smallest groups, and non‐significant results should not be interpreted as evidence of the absence of effect.

Gestational weight gain and duration of exclusive breastfeeding were considered clinically relevant variables. However, they were not available with adequate completeness and standardization for all participants, and for this reason, these variables were not included in the final adjusted models and were treated as potential sources of residual confounding in the interpretation of the results. Statistical analyses were performed using a 5% significance level (*p* < 0.05) and a 95% confidence interval. Data were analyzed using the Statistical Package for the Social Sciences (SPSS), version 31.0.

The IVAPSA project was approved by the Research Ethics Committee of the Hospital de Clínicas de Porto Alegre (HCPA; protocols no. 11‐0097 and 17‐0107) and by the Research Ethics Committee of the Grupo Hospitalar Conceição (GHC; protocol no. 11/027). The study was registered on the Brazilian National Research Ethics Platform (Plataforma Brasil) under the Certificate of Presentation for Ethical Consideration (CAAE) no. 65190217500005327. All procedures were conducted in accordance with the most recent guidelines and applicable regulations of the Brazilian National Health Council, Ministry of Health (Resolutions No. 466/2012 and No. 580/2018).

## 3 Results

In this study, 342 mother–child dyads were interviewed and allocated into five groups according to gestational characteristics: 55 in the DM group, 19 in the SAH group, 86 in the smoking group, 31 in the SGA‐defined impaired fetal growth group, and 151 in the control group. In Phase II, 115 mother–child dyads were reassessed at preschool age, corresponding to 33.6% of the initial sample. This reduction in sample size occurred due to mothers moving to other municipalities, changes in contact telephone numbers, inability to attend scheduled follow‐up visits, or refusal to continue participation in the study. The attrition rate from baseline to preschool follow‐up was substantial and may have affected the representativeness and precision of the estimates at later ages. Therefore, results referring to preschool age should be interpreted with caution, particularly in subgroup‐specific comparisons.

Table 1 shows the differences in sociodemographic and clinical characteristics of mothers, newborns, and preschool children distributed across the five groups with different adverse intrauterine conditions. Regarding maternal characteristics, there was a statistically significant difference in mean age (*p* = 0.004), with mothers in the DM group being older than those in the smoking group. The smoking group showed a higher proportion of single mothers and a lower proportion of married mothers compared with the other groups (*p* = 0.018). These mothers also had fewer years of schooling than those in DM, SGA, and control groups (*p* = 0.005) and had a lower family income than the control group (*p* = 0.001). Pre‐pregnancy BMI also differed among groups (*p* < 0.001): mothers in the DM group had a higher median pre‐pregnancy BMI than those in the SGA, control, and smoking groups, while mothers in the SGA group had a lower pre‐pregnancy BMI than those in the SAH group.

**TABLE 1:** Sociodemographic and clinical characteristics of mothers, newborns, and preschool children, distributed according to groups with different gestational clinical conditions and adverse intrauterine environments—IVAPSA Cohort Phases I and II (2011–2019), Porto Alegre (RS), Brazil.

	DM	SAH	Smoking	SGA	Control	Total	*p*
Maternal characteristics							
Maternal age, years (*n* = 341)	28.19 ± 5.94^a^	28.89 ± 7.78^ab^	24.66 ± 5.71^b^	24.26 ± 6.04^ab^	25.74 ± 7.01^ab^	25.9 ± 6.61	**0.004** ^**†**^
Number of children (*n* = 204)	2 (2–3)	2 (2–3.5)	2 (2–3)	2 (1.5–2.5)	2 (2–3)	2 (2–3)	0.438^‡^
Maternal race/skin color (*n* = 341)							0.795^§^
White	34 (63)	13 (68.4)	50 (58.1)	14 (45.2)	92 (60.9)	203 (59.5)	
Black	9 (16.7)	3 (15.8)	22 (25.6)	12 (38.7)	31 (20.5)	77 (22.6)	
Brown (parda)	10 (18.5)	3 (15.8)	11 (12.8)	4 (12.9)	26 (17.2)	54 (15.8)	
Others	1 (1.8)	0	3 (3.5)	1 (3.2)	2 (1.4)	7 (2.1)	
Marital status (*n* = 342)							**0.018** ^§^
Married	47 (85.5)	15 (78.9)	57 (66.3)*	27 (87.1)	127 (84.1)	273 (79.8)	
Single/divorced	8 (14.5)	4 (21.1)	29 (33.8)*	4 (12.9)	24 (15.9)	69 (20.2)	
Years of education (*n* = 337)	10 (8–11)^b^	9 (6.75–11)^ab^	8 (7–11)^a^	11 (8–11)^b^	10 (8–11)^b^	10 (8–11)	**0.005** ^**‡**^
Family income, BRL (*n* = 307)	1550 (1150–2625)^ab^	1200 (975–2075)^ab^	1227.5 (800–1950)^a^	1650 (1088.8–3000)^ab^	2000 (1200–2900)^b^	1660 (1000–2500)	**0.001** ^**‡**^
Pre‐pregnancy maternal BMI, kg/m^2^ (*n* = 306)	28.91 (24.83–32.05)^c^	27.24 (23.31–33.41)^bc^	23.81 (21.78–27.01)^ab^	21.28 (20.14–23.43)^a^	23.73 (20.7–27.01)^ab^	24.32 (21.41–28.03)	**< 0.001** ^**‡**^
Newborn characteristics							
Female sex (*n* = 342)	29 (52.7)	11 (57.9)	42 (48.8)	19 (61.3)	84 (55.6)	185 (54.1)	0.754^§^
Apgar score at 1 min (*n* = 339)	9 (8–9)	9 (8–9)	9 (8–9)	9 (8–9)	9 (8–9)	9 (8–9)	0.514^‡^
Apgar score at 5 min (*n* = 339)	9 (9–10)	10 (9–10)	10 (9–10)	10 (9–10)	9 (9–10)	10 (9–10)	0.086^‡^
Cesarean delivery (*n* = 342)	24 (43.6)*	12 (63.2)*	21 (24.4)	10 (32.3)	43 (28.5)	110 (32.2)	**0.005** ^**§**^
Birth weight, g (*n* = 340)	3484.18 ± 465.49^c^	3196.67 ± 441.38^bc^	3110.94 ± 404.71^b^	2562.29 ± 275.98^a^	3371.89 ± 440.82^c^	3241.72 ± 491.21	**< 0.001** ^**†**^
Preschool child characteristics							
Preschool age, months (*n* = 114)	49 (39–61)^a^	62 (52.25–65.75)^ab^	65.5 (60–68)^b^	64.5 (49.5–66.25)^ab^	60 (52.5–66)^b^	61 (51–66)	**0.001** ^‡^
Breastfeeding duration, months (*n* = 109)	8 (3–19)	25.5 (5.25–48)	8 (4–18)	13.5 (4.75–26.5)	18.5 (7.75–30)	14 (6–29)	0.138^‡^

*Note:* ^abc^ different letters denote differences between groups. Data are presented as mean ± standard deviation, median (interquartile range), or *n* (%). The bold values indicate statistical significance.

Abbreviations: BF: breastfeeding; BMI: body mass index; DM: diabetes mellitus; SAH: systemic arterial hypertension; SGA: small for gestational age, used in this study as a proxy for impaired fetal growth.

^†^ One‐way ANOVA with Bonferroni correction.

^‡^ Non‐parametric Kruskal–Wallis test with Dunn's post hoc test and Bonferroni adjustment.

^§^ Pearson's *χ* ^2^ test (* statistically significant association based on adjusted residuals at the 5% significance level).

Neonatal characteristics differed with respect to mode of delivery (*p* = 0.005), with the DM and SAH groups showing a higher proportion of cesarean sections than the other groups. Newborns in the SGA group had a lower mean birth weight than those in all other groups, whereas those in the smoking group had a lower mean birth weight than those in the DM and control groups (*p* < 0.001).

The longitudinal analysis indicated a significant effect of time on child growth, as assessed by the BMI‐for‐age z‐score (*p* < 0.001). A significant interaction between group and time was also observed (*p* < 0.001), as well as a three‐way interaction among group, time, and sex (*p* < 0.001), indicating that growth trajectories varied according to the type of intrauterine environment and the child's sex. However, the main effect of the group alone was not statistically significant (*p* = 0.528) (Table 2).

**TABLE 2:** Child growth according to BMI‐for‐age z‐scores, by sex and groups of different gestational clinical conditions with adverse intrauterine environments.

BMI‐for‐age z‐score	Group					GEE effects*			
	DM	SAH	Smoking	SGA	Control	Group	Time	Group × Time	Group × Time × Sex
	Mean ± SE	Mean ± SE	Mean ± SE	Mean ± SE	Mean ± SE	0.528	< 0.001	< 0.001	< 0.001
Female sex									
Birth	0.69 ± 0.19^b,f^	0.47 ± 0.27^bc,ef^	0.13 ± 0.17^c,e^	0.32 ± 0.49^bc,ef^	0.48 ± 0.13^b,ef^				
7 days	0.16 ± 0.20^a,ef^	−0.09 ± 0.19^a,ef^	0.24 ± 0.33^abcd,ef^	**−0.48 ± 0.29** ^ab,e^	0.36 ± 0.32^ab,f^				
15 days	0.44 ± 0.22^ab,f^	0.04 ± 0.47^ab,ef^	−0.07 ± 0.36^abc,ef^	−0.79 ± 0.32^a,e^	**0.51 ± 0.24** ^b,f^				
1 month	0.55 ± 0.45^abc,e^	−0.41 ± 0.44^a,e^	−0.42 ± 0.26^ab,e^	−0.42 ± 0.17^ab,e^	−0.02 ± 0.20^a,e^				
3 months	0.34 ± 0.32^ab,e^	−0.34 ± 0.56^ab,e^	−0.20 ± 0.22^a,e^	0.09 ± 0.43^abc,e^	−0.05 ± 0.23^a,e^				
6 months	0.85 ± 0.28^bc,f^	−0.27 ± 0.43^ab,e^	0.32 ± 0.33^cd,ef^	1.68 ± 0.16^d,g^	0.20 ± 0.19^ab,ef^				
Preschool age	1.70 ± 0.50^c,f^	1.24 ± 0.59^c,ef^	0.95 ± 0.40^d,ef^	**0.81 ± 0.20** ^c,ef^	0.36 ± 0.26^ab,e^				
Male sex									
Birth	0.67 ± 0.27^b,f^	−0.11 ± 0.53^a,ef^	0.28 ± 0.16^a,ef^	0.03 ± 0.15^b,e^	0.09 ± 0.18^ab,e^				
7 days	−0.00 ± 0.35^a,f^	−0.31 ± 0.16^a,f^	−0.54 ± 0.49^a,ef^	−1.52 ± 0.40^a,e^	0.07 ± 0.18^ab,f^				
15 days	0.46 ± 0.28^abc,e^	−0.18 ± 0.34^a,e^	−0.10 ± 0.23^a,e^	0.03 ± 0.16^b,e^	−0.12 ± 0.19^a,e^				
1 month	0.37 ± 0.26^abc,f^	−0.02 ± 0.51^a,ef^	−0.07 ± 0.26^a,ef^	−0.34 ± 0.59^ab,ef^	−0.27 ± 0.22^a,e^				
3 months	−0.37 ± 0.45^ab,ef^	−0.74 ± 0.31^a,e^	0.27 ± 0.32^a,f^	−0.08 ± 0.76^ab,ef^	−0.12 ± 0.25^a,ef^				
6 months	−0.84 ± 1.10^abc,ef^	−0.36 ± 0.62^a,e^	0.27 ± 0.42^a,ef^	1.52 ± 0.63^c,f^	0.17 ± 0.27^ab,e^				
Preschool age	1.28 ± 0.49^c,e^	0.58 ± 3.01^a,ef^	1.06 ± 0.96^a,ef^	2.64 ± 0.20^c,f^	0.86 ± 0.45^b,e^				

*Note:* Estimated effects by GEE—IVAPSA Cohort Phases I and II (2011–2019)—Porto Alegre (RS), Brazil. ^a,b,c,d^ identical letters do not differ according to the LSD test at the 5% significance level (within‐group comparisons); ^e,f,g^ identical letters do not differ according to the LSD test at the 5% significance level (between‐group comparisons). Bold indicates a significant difference between sexes within the same time point.

Abbreviations: BMI, body mass index; DM, diabetes mellitus; GEE, generalized estimating equations; LSD, least significant difference; SAH, systemic arterial hypertension; SE, standard error; SGA: small for gestational age, used in this study as a proxy for impaired fetal growth.

* Adjusted for mode of delivery, child sex and birth weight, maternal marital status, education level, age, parity, pre‐pregnancy BMI, and family income.

It was observed that the mean BMI‐for‐age z‐score of neonates at 7 days of life was lower than or equal to birth values in all groups (Table 2). At birth, female newborns in the DM group presented higher BMI‐for‐age z‐scores than those in the smoking group and reached elevated BMI‐for‐age z‐scores at preschool age (1.70 ± 0.50), differing from the control group (0.36 ± 0.26). Girls in the SAH group showed negative mean BMI‐for‐age z‐scores at most assessments; however, at preschool age, they reached a mean of 1.24 ± 0.59, which was significantly different from the other assessed ages, except for birth. In the smoking group, children maintained BMI‐for‐age z‐scores similar to those of the SGA group, differing only at 6 months of age, when girls in the SGA group showed a marked increase in BMI‐for‐age compared with previous assessments, reaching a mean z‐score of 1.68 ± 0.16, higher than that observed in the other groups.

Among boys, the DM group showed fluctuations in BMI‐for‐age z‐score values, starting with a mean BMI‐for‐age z‐score of 0.67 ± 0.27, becoming negative at three and 6 months of age, and returning to positive values at preschool age (1.28 ± 0.49) (Table 2). Children in the SAH group maintained negative z‐scores up to 6 months of age and, like the offspring of smoking mothers, did not show statistically significant intragroup differences over time. Boys in the SGA group showed a significant reduction in BMI‐for‐age z‐scores between the first and second assessments, with a mean of −1.52 ± 0.40 at 7 days. By 15 days of age, the BMI‐for‐age z‐score of these male newborns had already increased to 0.03 ± 0.16 and, at 6 months, to 1.52 ± 0.63, reaching 2.64 ± 0.20 at preschool age, a value higher than that observed in the DM and control groups.

Regarding child sex, a significant difference was observed (highlighted in bold in Table 2) in the SGA group, in which female newborns showed higher BMI‐for‐age z‐scores than male newborns at 7 days of life (*p* = 0.026) and, conversely, lower values at preschool age (*p* = 0.014). In the control group, female newborns had higher BMI‐for‐age z‐scores at 15 days of life compared with males. Figure 1 illustrates the BMI‐for‐age z‐scores of children from birth to preschool age in each of the different adverse intrauterine environment groups, according to sex.

**FIGURE 1:**
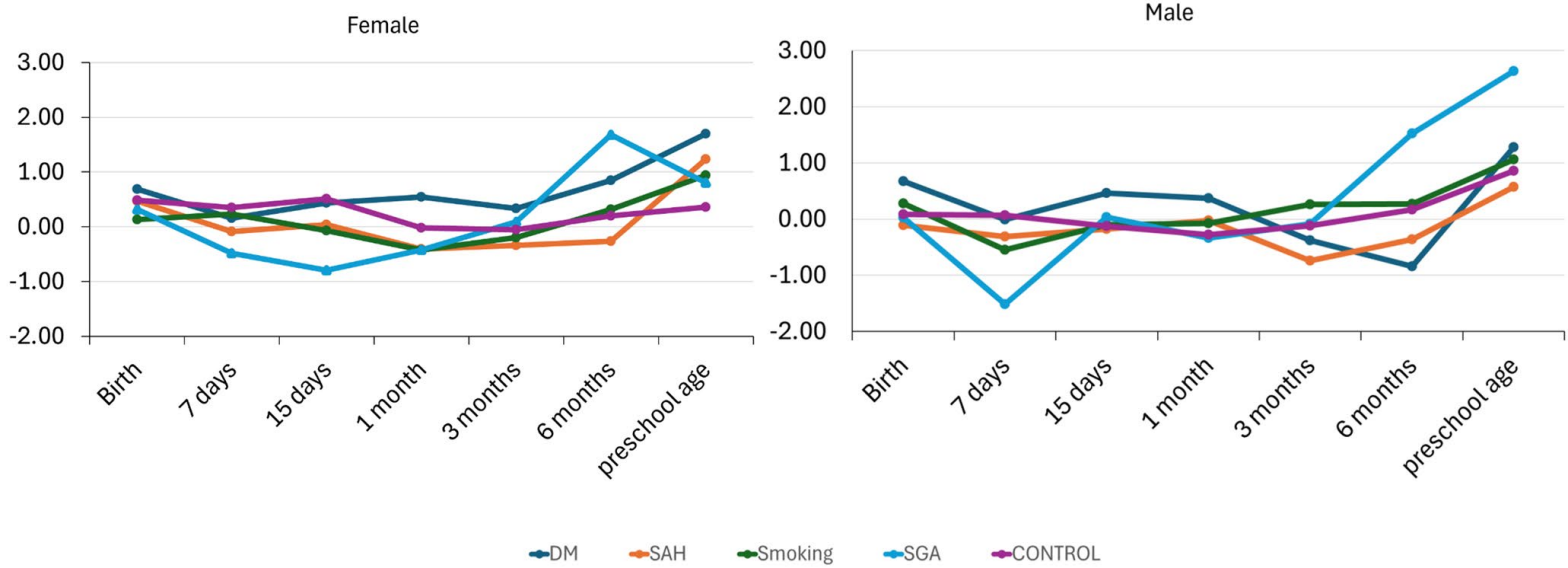
Growth analysis of infants and preschool children according to BMI‐for‐age z‐score, across different gestational clinical conditions with adverse intrauterine environments, using generalized estimating equations (GEE). IVAPSA Cohort, Phases I and II—Porto Alegre (RS), Brazil. BMI: Body mass index for age; DM: Diabetes mellitus; SAH: Systemic arterial hypertension; SGA: Small for gestational age, used in this study as a proxy for impaired fetal growth.

## 4 Discussion

The present study showed that BMI‐for‐age trajectories from birth to preschool age differed according to the intrauterine environment, with significant interactions between exposure group, time, and child sex. Although the findings should be interpreted cautiously because of the unequal subgroup sizes and substantial loss to follow‐up, they suggest that adverse intrauterine environments may influence postnatal growth in distinct ways. The most evident patterns were the persistently higher BMI‐for‐age among girls exposed to maternal DM and the marked catch‐up growth observed in children born SGA‐defined impaired fetal growth, particularly among boys.

Differences in maternal characteristics across exposure groups may have contributed to these growth patterns. Mothers in the smoking group had lower family income, fewer years of schooling, and a lower proportion of mothers living with a partner, which may indicate greater difficulties in providing adequate childcare, in addition to the well‐established harms of smoking to the fetus and the child. Pre‐pregnancy BMI was highest in the DM group and lowest in the SGA group, findings consistent with previous studies (DeFronzo et al. 2015; Yang et al. 2023). Because maternal pre‐pregnancy BMI is an important determinant of offspring adiposity (Mao et al. 2024), these differences reinforce the importance of considering maternal nutritional status when interpreting early childhood growth trajectories.

In the present study, a reduction or maintenance of BMI‐for‐age z‐score values from birth to 7 days of life was observed across all groups. This finding was expected, as newborns typically experience a weight loss of approximately 7%–8% during the first days of life due to the loss of free extracellular water (DiTomasso and Cloud 2019) and may take 7–10 days to regain their birth weight (Sarmiento‐Aguilar et al. 2022; Tigabu et al. 2025).

The difference in BMI‐for‐age z‐scores between sexes was observed at two time points in the SGA group and at one‐time point in the control group. Regarding potential sex‐related differences, Diaz et al. (2020), in a prospective cohort study conducted in the United States, reported that male newborns had greater birth weight and length than girls; however, BMI‐for‐age z‐scores did not differ between sexes at birth. A study assessing preterm newborns found similar results for birth weight and length, but in this case, BMI‐for‐age was also higher among boys (Shoji et al. 2020).

The mean BMI‐for‐age z‐score at birth among male newborns in the SGA group was lower than that observed in the DM group. In contrast, female newborns in the DM group had higher mean BMI‐for‐age z‐scores at birth than those in the smoking group. Lower mean values in the SGA group were expected, considering the possible growth restriction that occurred during the intrauterine period (Narang et al. 1997), which leads to reduced fetal growth velocity (Yadav and Rustogi 2015). Bartels et al. (2020) reported that fetal growth velocity increased with higher maternal fasting glucose levels. This finding may have influenced BMI‐for‐age at birth among newborns in the DM group in the present study, as glycemic control is impaired in this condition.

At preschool age, female infants born to mothers with DM had a high mean BMI‐for‐age z‐score, which was statistically higher than that observed in the control group. Considering that this group also presented the highest mean values at birth, these findings are consistent with a study reporting higher BMI‐for‐age percentiles at 5 years of age among children with greater fetal weight gain (Bartels et al. 2020). A possible explanation is the impact of intrauterine exposure to DM, which has been shown to confer a higher risk for the development of obesity in the offspring than genetic predisposition alone (Dabelea et al. 2000).

In both sexes, infants in the SGA group, despite starting with lower BMI‐for‐age z‐scores, reached higher BMI‐for‐age values as early as 6 months of age. This pattern may be explained by catch‐up growth, which commonly occurs up to the first 2 years of life (Adam‐Raileanu et al. 2025). Conversely, rapid and excessive growth up to 2 years of age and particularly within the first 4 months of life is an important risk factor for overweight and obesity between 2 and 5 years of age among infants born SGA (Li et al. 2022). It is well established that this accelerated growth represents a compensatory mechanism for fetal growth restriction and is observed in approximately 90% of SGA newborns (Yadav and Rustogi 2015). Our findings reinforce the importance of monitoring growth in this population beyond infancy, particularly among children exhibiting accelerated weight gain during the first months of life.

Children in the SAH group generally presented lower BMI‐for‐age z‐scores throughout early childhood, although positive values were observed at preschool age. Previous studies have associated hypertensive disorders of pregnancy with impaired fetal growth and increased risk of SGA births (Bakker et al. 2011), whereas a retrospective cohort study of adolescents found that maternal SAH was significantly associated with overweight/obesity in adolescence (Kuciene and Dulskiene 2022). Because our follow‐up ended at preschool age, the long‐term implications of these trajectories remain uncertain.

Similarly, children exposed to maternal smoking had relatively low birth weight followed by increasing BMI‐for‐age during follow‐up, a pattern that agrees with previous reports linking prenatal smoking to reduced fetal growth (Lev‐Ari et al. 2020; Rogers 2019) and greater risk of childhood overweight (Perkins et al. 2023). However, smoking exposure was classified only as a binary variable, preventing the evaluation of dose‐dependent effects or differences according to smoking intensity or timing during pregnancy.

The observed growth trajectories are biologically plausible within the Developmental Origins of Health and Disease (DOHaD) framework. Hyperglycemia during pregnancy may promote fetal overnutrition and increased adiposity (Bartels et al. 2020), whereas placental insufficiency associated with hypertensive disorders may restrict fetal growth (Pinheiro et al. 2016). In pregnancies complicated by smoking, fetal hypoxia, and epigenetic alterations may contribute to compensatory postnatal growth (Rogers 2019; White et al. 2022), while children born SGA frequently undergo rapid catch‐up growth following fetal nutrient restriction (Adam‐Raileanu et al. 2025; Li et al. 2022). Although these mechanisms differ, they may converge toward altered metabolic programming and increased susceptibility to obesity later in life.

Among the limitations of this study, the substantial attrition between baseline and preschool follow‐up should be acknowledged, as it may have introduced attrition bias and reduced statistical precision, particularly in the smaller SAH and SGA groups. Although GEE models allowed the inclusion of all available repeated measurements, the results should be interpreted with caution because missing data may not have occurred completely at random. Additional limitations include the classification of maternal smoking as a binary exposure, without information on smoking intensity, timing, or cessation during pregnancy, precluding dose–response analyses. Furthermore, gestational weight gain and exclusive breastfeeding duration could not be included in the adjusted models because of incomplete data, leaving the possibility of residual confounding. Finally, different types of diabetes were analyzed as a single exposure group despite their potentially distinct effects on offspring growth.

Despite these limitations, this study has important strengths. It evaluated children exposed to several clinically relevant adverse intrauterine environments within the same longitudinal cohort, using standardized anthropometric measurements collected at seven time points from birth to preschool age. This design enabled the comparison of growth trajectories across different gestational conditions while including a control group, providing a broader perspective on the potential influence of prenatal exposures on early childhood growth.

## 5 Conclusions

Different adverse intrauterine environments were associated with distinct BMI‐for‐age trajectories from birth to preschool age, with sex‐specific patterns observed for some exposure groups. These findings reinforce the importance of considering prenatal exposures when monitoring early growth and may help identify children who could benefit from targeted follow‐up during early childhood.

## Author Contributions

**Gabriela Koglin:** formal analysis, visualization, writing – original draft preparation, writing – review and editing. **Clécio Homrich da Silva:** conceptualization, methodology, project administration, supervision, writing – review and editing. **Denise Ruschel Bandeira:** writing – review and editing. **Nadia Cristina Valentini:** writing – review and editing. **Marcelo Zubaran Goldani:** conceptualization, funding acquisition, methodology, project administration, supervision, writing – review and editing. **Juliana Rombaldi Bernardi:** conceptualization, formal analysis, funding acquisition, investigation, methodology, supervision, writing – review and editing. All authors read and approved the final manuscript.

## Funding

This study was funded by Fundação de Amparo à Pesquisa do Estado do Rio Grande do Sul (FAPERGS)/National Council for Scientific and Technological Development (CNPq) grant 10/0018.3 (National Council for Scientific and Technological Development), National Program of Support to Centers of Excellence/Programa de Apoio a Núcleos de Excelência (PRONEX 2009) (Program to Support Centers of Excellence), FIPE (Fundo de Incentivo à Pesquisa e Eventos)/HCPA (Research Incentive Fund of the Hospital de Clínicas de Porto Alegre), and CAPES (Coordination for the Improvement of Higher Education Personnel). The funding institutions reviewed the study proposal and provided laboratory space and equipment, data analysis support, and assistance in manuscript development.

## Ethics Statement

The IVAPSA project was approved by the Research Ethics Committees of the Hospital de Clínicas de Porto Alegre (No. 11‐0097 and 17‐0107) and the Grupo Hospitalar Conceição (No. 11/027). The study was registered on Plataforma Brasil (CAAE No. 65190217500005327). All procedures were conducted in accordance with Brazilian National Health Council Resolutions No. 466/2012 and No. 580/2018. Written informed consent was obtained from the participants, and parents or legal guardians provided consent for their children's participation, as applicable.

## Conflicts of Interest

The authors declare no conflicts of interest.

## Data Availability

If requested, the authors will provide the data or will cooperate fully in obtaining and providing the data on which the manuscript is based for examination by the editors or their assignees.
